# Beyond the Plate: Patient Perspectives on Diet and Daily Life with Crohn’s Disease—A National Survey

**DOI:** 10.3390/jcm14165648

**Published:** 2025-08-09

**Authors:** Sarah Bencardino, Ferdinando D’Amico, Ambra Ciliberto, Silvio Danese

**Affiliations:** Gastroenterology and Endoscopy, IRCCS Ospedale San Raffaele and Vita-Salute San Raffaele University, 20132 Milan, Italy; bencardino.sarah@hsr.it (S.B.); damico.ferdinando@hsr.it (F.D.); ciliberto.ambra@hsr.it (A.C.)

**Keywords:** Crohn’s disease, dietary habits, psychological impact, patient management, disease remission and flare-ups

## Abstract

**Background:** Crohn’s disease (CD) is a chronic inflammatory bowel disease that significantly affects patients’ quality of life. Nutrition is increasingly recognized as a modifiable factor influencing disease activity and symptom management. Despite growing interest, structured dietary guidelines for CD are lacking, and patients often rely on personal experience or fragmented advice. This study aimed to investigate patients’ perceptions of diet, the support they receive, and the psychosocial burden of dietary management in CD. **Methods**: A nationwide online survey was conducted in Italy from April to May 2025 among individuals diagnosed with CD. The questionnaire, developed in line with the CROSS reporting guidelines, comprised 30 multiple-choice questions across five sections: demographics, disease characteristics, dietary habits during remission, dietary habits during flare-ups, and psychological impact. Invitations were distributed through patient associations, webinars, and gastroenterology professionals. Responses were anonymized. **Results:** A total of 222 participants completed the survey (59.5% female, most aged 30–39 years). Fatigue was the most common symptom (71.6%), frequently persisting even during remission. Nearly half of respondents reported diet as “very important” in disease management, yet only 32% had received a formal referral to a nutritionist. The most commonly adopted dietary approach was a low-fiber diet, while awareness of evidence-based protocols like the Crohn’s disease exclusion diet (CDED) was limited (11.7%). Social and psychological burdens were significant, with 79.2% reporting anxiety when outside their home. **Conclusions**: Dietary education and psychological support are unmet needs for CD patients. Improved access to tailored nutritional counseling and greater awareness of validated dietary approaches may enhance disease management and quality of life.

## 1. Introduction

Crohn’s disease (CD) is a chronic inflammatory condition of the gastrointestinal tract that often begins in young adulthood and can have a profound impact on quality of life (QoL). Alongside medications and non-medical therapies, many patients are interested in how lifestyle factors—particularly diet—may influence their symptoms and disease course. Over the past years, there has been increasing scientific interest in the role of nutrition in modulating intestinal inflammation, maintaining remission, and improving overall health outcomes in CD [1].

Despite this growing body of research, many questions remain unanswered, and there are currently no universally accepted dietary guidelines tailored specifically to individuals with CD. Current guidelines recommend an individualized dietary approach, aiming to maintain nutritional adequacy and quality of life [2]. Exclusive enteral nutrition (EEN) is endorsed as first-line induction therapy in pediatric patients but not routinely in adults, for whom a balanced, whole-food diet—such as the Mediterranean diet—is preferred [2]. Structured interventions like the CD exclusion diet (CDED) may be considered in mild-to-moderate cases or when EEN is not tolerated, as they are easier to follow and support social participation, while unnecessarily restrictive diets should be avoided to prevent deficiencies and social limitations. However, in everyday life, patients are often left to navigate dietary decisions on their own, experimenting with different foods or restrictive diets in an effort to manage symptoms, prevent flare-ups, or support medical treatments.

In parallel, the recent implementation of patient self-reported outcome measures (PROMs) in the clinical assessment of IBD patients has raised awareness of symptoms such as fatigue, persistent mental and/or physical exhaustion, weakness, and tiredness, highlighting the importance of incorporating patient perspectives into both clinical care and research [3]. However, the experiences and perspectives of patients remain underrepresented in clinical research.

The aim of this survey is to explore how patients with CD perceive the role of diet in managing their condition. We are particularly interested in understanding which dietary approaches patients follow, whether they have received professional nutritional guidance, what challenges they face when making food choices, and how these dietary strategies impact their well-being. By collecting this information, we hope to identify common practices, unmet needs, and potential areas where nutritional support could be improved.

## 2. Materials and Methods

This survey was designed to gather information from patients with a confirmed diagnosis of CD. The survey was conducted from April to May 2025 using an online platform. The study was conducted and reported in compliance with the Consensus for Reporting of Survey Studies (CROSS) guidelines [4]. A CROSS checklist is provided as Supplementary Material. Survey invitations were disseminated through multiple channels, including the hospital mailing lists of all adult patients with a confirmed diagnosis of CD, as recorded in the institutional database. Screening questions at the start of the survey ensured that only eligible participants were included. To prevent duplicate entries, email registration was required, while responses remained anonymous. Participants granted permission for data collection at the beginning of the survey. The questionnaire consisted of 30 multiple-choice items organized into five sections. Two sections addressed demographics and disease characteristics, while another two sections explored dietary and nutritional aspects of CD, with distinctions between remission and flare phases. The final section investigated the psychological dimension, assessing the emotional and social impact of the disease on daily life. For transparency, the number of respondents per question was reported to account for missing data. The study followed the principles of the Declaration of Helsinki. As the survey was entirely non-interventional and not designed to inform clinical management, formal ethics approval and written informed consent were not required. All data were fully anonymized [5].

## 3. Results

### 3.1. Demographics

In total, 222 patients from Italy participated in the survey. The most represented place was the Northern Italy (143/222, 64.41%), followed by Southern Italy (53/222, 23.87%) and Central Italy (26/222, 11.71%). The most represented age was between 30 and 39 years (58/222, 26.13%), and most participants were female (132/222, 59.46%) (Table 1).

### 3.2. Characteristics of the Disease

**Figure 1:**
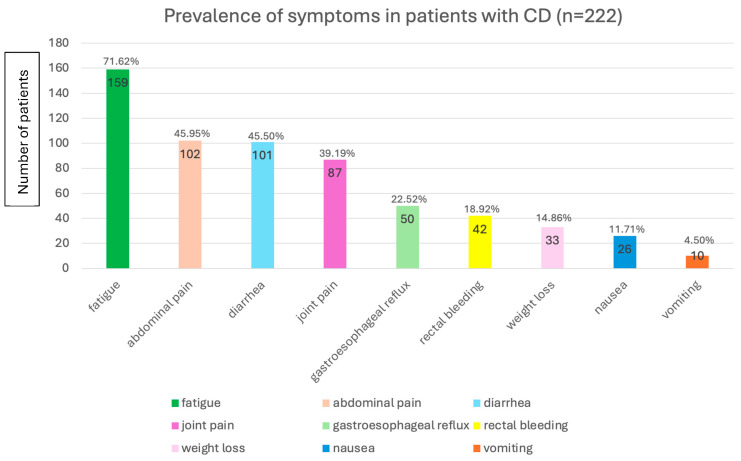
Prevalence of symptoms in patients with Crohn’s disease. CD: Crohn’s disease; n: number of patients.

The majority of participants (82/222, 36.94%) reported being diagnosed between the ages of 20 and 29, followed by those diagnosed between 10 and 19 years of age (51/222, 22.97%). Most participants (181/218, 83.03%) reported an ileal involvement of disease. The most frequently reported symptom was fatigue (defined as a sense of unusual or abnormal tiredness, exhaustion, or weakness) (159/222, 71.62%). Among those who reported fatigue, the majority experienced it persistently (103/159, 64.78%) and stated that fatigue was present even when the disease was in remission (99/159, 62.26%). Other frequent symptoms were abdominal pain (102/222, 45.95%) and diarrhea (101/222, 45.50%), indicating a high prevalence of core gastrointestinal symptoms (Figure 1). Most patients (199/222, 89.64%) reported being on pharmacological therapy. Among them, the majority were receiving biologic therapy (153/199, 76.88%).

### 3.3. Role of Diet

**Figure 2:**
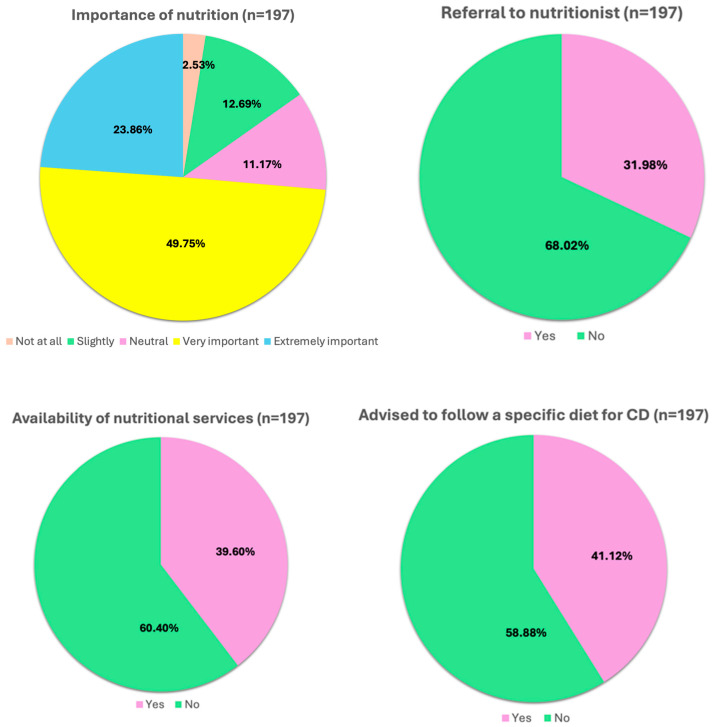
Role of diet and clinical nutrition support. CD: Crohn’s disease; n: number of patients.

Participants were asked to evaluate the importance they attributed to nutrition in managing CD and whether they believed diet can influence disease activity. Of the 197 individuals who responded, the majority recognized a significant role of nutrition: 98 (98/197, 49.75%) considered it very important, and 47 (47/197, 23.86%) rated it as extremely important. A smaller group expressed a neutral opinion (22/197, 11.17%), while fewer individuals found it to be of little (25/197, 12.69%) or very little importance (5/197, 2.53%) (Figure 2). The majority (117/197, 59.39%) were informed about the potential impact of diet on disease activity by their gastroenterologist. However, less than half of the patients (63/197, 31.98%) were formally referred to a nutritionist, most commonly within a hospital setting (42/63, 66.67%). Notably, among those who had not received any recommendation (134/197, 68.02%), a considerable proportion (83/134, 61.94%) independently sought a private nutrition consultation, underscoring patient-driven interest in dietary support. Regarding the availability of nutritional services, 78 patients (78/197, 39.60%) reported that a dietitian or nutritionist was accessible through a facilitated pathway at their care center, while the remaining 119 (119/197, 60.40%) indicated that no such support was available. When asked whether they had ever been advised to follow a specific diet for CD, the majority (116/197, 58.88%) had not received any targeted dietary guidance, while 81 participants (81/197, 41.12%) responded affirmatively. Similarly, 72 respondents (72/197, 36.55%) reported being advised to use nutritional supplements. Among them, 25 (25/72, 34.72%) were recommended supplements formulated specifically for CD. Other recommendations included high-calorie and/or high-protein supplements (11/72, 15.28%), vitamin supplementation (43/72, 59.72%), and omega-3 fatty acids (12/72, 16.67%). Despite these interventions, most patients (125/197, 63.45%) had never received advice on nutritional supplementation as part of their disease management.

#### 3.3.1. Perceived Role of Nutrition and Daily Management in the Remission Phase

Participants were asked to identify which meal of the day they found most challenging to organize in their typical daily routine. Out of 185 respondents, the most frequently reported difficulty concerned lunch (78/185, 42.16%), often due to work-related constraints. Participants were asked whether they had ever had to give up social occasions—such as dinners, work lunches, or outings with friends—because of their condition. Of the 184 individuals who responded, the majority (130/184, 70.65%) answered yes, indicating that CD had negatively impacted their social participation. Participants were asked whether they follow a specific diet for CD. The majority (122/183, 66.7%) indicated that they do not follow a special diet, while a smaller group (61/183, 33.3%) reported adhering to a specific dietary regimen to manage their condition. Among those following a diet, the largest group (53/61, 86.9%), followed a low-fiber diet, three (3/61, 4.9%) were on the CDED, two (2/61, 3.3%) adhered to a high-protein, high-calorie diet, and the remaining three participants (3/61, 4.9%) did not specify their dietary plan. Interestingly, most of the respondents (161/183, 88%) did not know what CDED was, highlighting the low awareness of this diet among patients.

#### 3.3.2. Perceived Role of Nutrition and Daily Management During Flare-Ups

Participants were asked the same set of questions during different stages of disease activity. Notably, the responses remained consistent between flare-ups and remission. The majority (73/165, 44.2%) found lunch to be the most challenging meal to plan. Consistently, more than two-thirds of the patients (119/162, 73.5%) reported that they had to forgo social occasions due to their condition. Finally, when asked whether they follow a specific diet for CD, the majority (104/162, 64.2%) did not follow any particular dietary regimen, while 58 (58/162, 35.8%) reported adhering to a special diet. Of the 58 individuals who reported following a diet, the majority (38/58, 65.5%) followed a low-fiber diet, 5 (5/58, 8.6%) were on the CDED, 2 (2/58, 3.4%) adhered to a high-protein, high-calorie diet, and the remaining participants did not specify their dietary plan. Once again, awareness of the CDED was notably low; most of the patients (144/163, 88.34%) reported not being familiar with this evidence-based dietary approach.

### 3.4. Psychological Impact: Navigating the Mental Health Challenges of Crohn’s Disease

**Figure 3:**
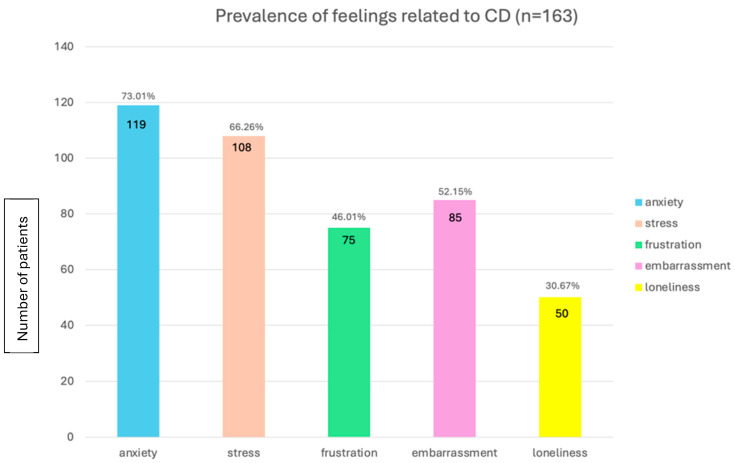
Prevalence of feelings related to Crohn’s disease. CD: Crohn’s disease; n: number of patients.

Regarding feelings and emotions, the majority (129/163, 79.2%) reported feeling anxious about spending the day outside of their home, at work, or at university. Morever, almost two-thirds of patients (105/163, 64.4%) reported that the disease limited their activities such as sports or other hobbies. Anxiety was the prevalent emotion related to the disease (119/163, 73%), followed by stress (108/163, 66.3%), frustration (85/163, 52.1%), embarrassment (75/163, 46%), and loneliness (50/163, 30.7%) (Figure 3). Participants were asked how much of their free time they dedicate to following the most suitable dietary regimen for their condition, including activities such as shopping, cooking, researching, or monitoring symptoms. Around one-third (70/163, 42.9%) reported spending less than 2 h per week on these tasks. This was followed by 50 (50/163, 30.7%) who spend between 2 and 4 h per week on this, 23 (23/163, 14.1%) who spend more than 6 h per week, and 20 (20/163, 12.3%) who dedicate more than 4 h per week to this. Regarding their sources of information about the disease, the majority (93/163, 57.1%) rely on their doctor for information, 54 (54/163, 33.1%) turn to online social platforms, 11 (11/163, 6.7%) use patient associations, and 5 (5/163, 3.1%) indicated other sources.

## 4. Discussion

This survey provides a multifaceted view of the perspective of 222 patients with CD across Italy, providing valuable insights into dietary management and psychosocial burden.

Symptoms remain a substantial burden, even in patients currently under treatment. These findings are consistent with the results of the PODCAST study, a large, non-interventional, cross-sectional analysis that sheds light on the considerable burden still faced by patients with IBD [6]. Despite advances in therapeutic strategies, a substantial proportion of individuals continue to experience suboptimal disease control, predominantly driven by impaired QoL.

Among the symptoms, fatigue emerged as the most frequently reported, affecting over 70% of participants, and was often described as persistent and present even during remission phases. This is in line with several studies reporting fatigue as a common symptom among individuals with IBD with prevalence rates ranging from 41% to 61% [7,8,9,10,11]. Despite its frequency, the underlying causes of fatigue in IBD remain poorly understood. Its persistence even in patients in remission suggests that inflammation alone does not fully account for it. Other contributing factors under investigation include anemia, malnutrition-related micronutrient deficiencies, side effects of medications, sleep disturbances, and alterations in the gut microbiota [12,13]. Among the potential strategies to alleviate fatigue, particular attention should be given to identifying and correcting nutritional deficiencies—such as iron, vitamin B12, folate, and vitamin D—which are common in CD patients and may significantly contribute to persistent fatigue, even during remission [14].

Dietary management was widely recognized by patients as a critical component of disease control. Nearly three-quarters of respondents believed that nutrition plays a “very” or “extremely” important role in managing CD, yet more than half had not received any formal dietary guidance. Only 32% had been referred to a nutritionist, suggesting a significant gap between patient needs and the availability of multidisciplinary care. Interestingly, among those not referred, a large proportion independently consulted a private dietitian—demonstrating strong patient initiative but also highlighting shortcomings in the current healthcare infrastructure. To address this gap, future efforts should focus on integrating structured nutritional counseling into routine care pathways, ensuring timely referral to trained dietitians as part of a multidisciplinary approach to CD management.

Adherence to specific dietary regimens was relatively low, with just 33.3% of patients reporting any form of dietary modification. Among these, the vast majority followed a low-fiber diet (with a reduction in overall fiber intake but no specification of the type of fiber restricted), while very few adhered to more structured evidence-based regimens such as the CDED. However, the use of a low-residue (minimizing the amount of undigested material reaching the colon, focusing on limiting foods that increase stool volume and frequency) or low-fiber diet during active IBD is supported by limited scientific evidence [15]. Although patients often reduce food intake during flare-ups—partly due to inflammation-related loss of appetite—and frequently report that eating worsens abdominal pain and bowel movements, the actual benefits of fiber restriction remain unclear [16]. A randomized controlled trial involving 71 patients, most of whom had active CD, found no significant difference in disease activity or symptoms between those on a low-fiber diet and those following an unrestricted diet [17]. These findings suggest that strict fiber restriction may not be necessary for symptom management during active disease, except for patients with strictures. Current dietary and nutritional strategies for managing active CD, such as EEN and partial enteral nutrition (PEN) combined with CDED [18], while limiting insoluble fiber, are not strictly low-residue diets; rather, they involve the use of specialized formulas that often contain dairy proteins and easily absorbable nutrients [18]. Therefore, although these protocols reduce fiber intake, they differ fundamentally in composition and purpose from general fiber restriction or low-residue diets. All of these nutritional therapies influence the composition and activity of the gut microbiota, and this effect on the microbiome—rather than merely reducing stool volume—may primarily contribute to symptom improvement and decreased intestinal inflammation [1]. Current ECCO guidelines and emerging evidence highlight the potential role of dietary interventions in the management of CD, particularly in selected and motivated patients [19]. EEN may be considered as an induction therapy for adult patients with mild-to-moderate disease who prefer to avoid corticosteroids and have access to appropriate dietetic support [19]. While no single dietary approach has been shown to benefit all patients, nutritional strategies should be individualized based on disease activity, patient preferences, and resource availability. Additionally, PEN may be useful in maintaining remission in a subset of patients who can tolerate it, either as monotherapy or alongside pharmacologic treatment [19]. Moreover, in line with these recommendations and the growing interest in integrated therapeutic approaches, several ongoing clinical studies are currently investigating the efficacy of combining dietary interventions with pharmacologic therapy in CD. For instance, trials are assessing the impact of CDED in combination with biologic agents such as anti-TNF therapies or ustekinumab, aiming to enhance clinical remission and reduce mucosal inflammation [20,21]. Other studies are exploring specific dietary protocols in patients already receiving pharmacologic treatment, suggesting a potential synergistic effect between diet and medication in controlling disease activity [22]. These emerging data support the notion that dietary strategies should not be viewed as alternative therapies, but rather as integral components of a comprehensive treatment plan for CD. Overall, access to dietary counseling should be considered an essential component of care, especially during disease flares. Despite these recommendations, in our survey, 60.4% of patients reported that no such support was available, and 58.9% stated they had not received any targeted dietary guidance. These findings underscore a disconnect between guideline-based care and real-world patient experience, suggesting an urgent need to implement structured nutritional counseling as a standard component of CD management.

As part of the growing emphasis on dietary strategies in clinical recommendations, a recent pilot randomized controlled trial involving adults with mild-to-moderate active disease demonstrated that the CDED, used either alone or combined with PEN as monotherapy, achieved a 62% remission rate at week 6 [23]. Furthermore, 50% of patients sustained remission through week 24, and 35% reached endoscopic remission. Therefore, according to current evidence, the recent ESPEN guidelines suggest that the CDED may be considered as an alternative to exclusive EEN in adults with mild-to-moderate CD [24]. However, awareness of the CDED was remarkably low, with only 11.7% of participants reporting familiarity with it—highlighting a major educational gap. This lack of awareness was consistent across both remission and active disease phases. This limited awareness likely reflects variable clinician knowledge, limited dissemination outside tertiary IBD centers, and the scarce availability of structured nutritional counseling. Despite the burden of dietary self-management, most patients spend fewer than four hours per week on meal planning, cooking, and monitoring symptoms—possibly reflecting either a coping strategy or barriers such as fatigue, limited resources, or lack of professional guidance. This limited awareness of CDED, despite its growing support in clinical guidelines and evidence, combined with the minimal time patients dedicate to dietary self-management, underscores the practical challenges many face in translating dietary recommendations into daily routines. Enhancing CDED awareness will require better integration of dietitians in IBD care, targeted clinician education, and accessible patient-focused resources.

These findings not only highlight the gap between recommended dietary strategies and real-world practice but also reveal how the cumulative burden of symptom persistence, limited dietary support, and daily organizational challenges can significantly exacerbate the psychological impact of CD. Nearly 80% of respondents reported experiencing anxiety when away from home, and 64% noted limitations in pursuing leisure activities. Anxiety, stress, and frustration were the most commonly reported emotions, underscoring the chronic mental strain associated with the condition. Our findings are consistent with the psychological burden reported in the IBD literature, where validated PROMs such as Hospital Anxiety and Depression Scale (HADS) and IBD Questionnaire (IBDQ) have similarly highlighted high rates of anxiety and impaired quality of life among patients [25]. Interestingly, data from the medical literature suggest that the prevalence of anxiety in other chronic conditions, including diabetes, metastatic cancer, and coronary artery disease, is generally lower than that observed in CD, with rates of approximately 15% for anxiety and 12.3% for depression in the general adult population with chronic illnesses [26]. Furthermore, a shorter disease duration correlates with increased anxiety and depressive symptoms, especially among adolescents and young adults, indicating that individuals in the early stages of the disease are more susceptible [27]. These data suggest that psychological support should be considered a core component of comprehensive CD care. Patients who adhere to their treatment plans and attend regular follow-up appointments tend to report a higher QoL compared to those who do not [28]. These findings highlight the multifaceted approach needed for effective CD management, where psychological support plays a crucial role alongside other modifiable factors such as dietary habits, which have also been shown to significantly influence both symptom control and QoL.

This study provides valuable insight into the real-life setting of Italian CD patients, highlighting gaps in dietary support and psychosocial care. Its strengths include a large sample size, a questionnaire specifically designed to investigate the role of nutrition, and adherence to CROSS guidelines. However, limitations include reliance on self-reported data and a lack of correlation with clinical, biochemical, and endoscopic data. Moreover, while this survey aimed to capture a national perspective, the higher participation from Northern Italy relative to Central and Southern regions should be acknowledged as a potential limitation when interpreting the representativeness of the findings. The future management of CD increasingly emphasizes the critical role of diet as a modifiable factor influencing disease activity and QoL. Continuous and precise monitoring of dietary habits is essential to tailor nutritional interventions that can help control symptoms and maintain remission. The development of innovative digital tools and mobile applications for real-time diet tracking will be instrumental in supporting both patients and healthcare providers in optimizing individualized dietary strategies.

Furthermore, telemedicine offers promising opportunities to enhance dietary management by enabling remote consultations, continuous monitoring, and timely dietary adjustments based on patients’ evolving needs [29]. This approach not only improves patient engagement and adherence but also facilitates the integration of dietary data with clinical and psychological assessments.

In addition, emerging research on the gut microbiota highlights the potential to modulate the intestinal flora through diet-based interventions or adjunctive therapies such as fecal microbiota transplantation, aiming to restore microbial balance and reduce inflammation [30]. This integrated strategy may open new avenues for controlling disease progression and improving long-term outcomes in CD.

## 5. Conclusions

This survey highlights the multifaceted burden of CD, extending beyond clinical symptoms to deeply impact dietary habits, psychological well-being, and social participation. Despite advances in pharmacologic therapies and growing evidence supporting dietary and psychological interventions, substantial gaps persist in real-world care. Limited access to structured nutritional counseling, low awareness of evidence-based dietary strategies, and insufficient psychological support reflect a disconnect between clinical guidelines and patient experience. Bridging these gaps through multidisciplinary, patient-centered approaches is essential to improving disease management and enhancing QoL for individuals with CD.

## Figures and Tables

**Table 1:** Demographic characteristics of patients and characteristics of the disease.

**Patient (n = 222)**	
**Sex**	
Male	89 (40.09%)
Female	132 (59.46%)
Others	1 (0.45%)
**Age (years)**	
18–19 y	9 (4.05%)
20–29 y	57 (25.68%)
30–39 y	58 (26.13%)
40–49 y	47 (21.17%)
>50 y	51 (22.97%)
**Place of residence**	
Northern Italy	143 (64.41%)
Central Italy	26 (11.71%)
Southern Italy	53 (23.87%)
**Age at diagnosis (years)**	
1–9 y	2 (0.90%)
10–19 y	51 (22.97%)
20–29 y	82 (36.94%)
30–39 y	46 (20.72%)
40–49 y	29 (13.06%)
>50 y	12 (5.41%)
**Location**	
Ileal	181/218 (83.03%)
Colonic	69/218 (31.65%)
Perianal	28/218 (12.84%)
Gastro-duodenal	15/218 (6.88%)
**Symptoms**	
Fatigue	159 (71.62%)
Abdominal pain	102 (45.95%)
Diarrhea	101 (45.50%)
Joint pain	87 (39.19%)
Gastroesophageal reflux	50 (22.52%)
Rectal bleeding	42 (18.92%)
Weight loss	33 (14.86%)
Nausea	26 (11.71%)
Vomiting	10 (4.50%)
**Therapy**	
Biologic therapy	153/199 (76.88%)
Systemic corticosteroids	16/199 (8.04%)
Mesalamine	19/199 (9.55%)
Experimental trial protocols	2/199 (1.01%)
Small-molecule drugs	3/199 (1.51%)
Budesonide	2/199 (1.01%)
Immunosuppressants	3/199 (1.51%)
Not specified	1/199 (0.50%)

## Data Availability

The data that support the findings of this study are available from the corresponding author upon reasonable request.

## Supplementary Materials

The following supporting information can be downloaded at https://www.mdpi.com/article/10.3390/jcm14165648/s1.
