# Supplementary material for: Beyond the Plate: Patient Perspectives on Diet and Daily Life with Crohn’s Disease—A National Survey
Source: J Clin Med. 2025 Aug 9;14(16):5648. doi: 10.3390/jcm14165648 (PMC12387096; doi:10.3390/jcm14165648)
Supplement: Supplementary file 1 [file jcm-14-05648-s001.zip › jcm-3774603-supplementary.pdf]

Supplement Data

1. Questionnaire (Italian version)

Profilo rispondenti

1. **In che fascia d'età ti collochi?***
   - 1-9
   - 10-19
   - 20-29
   - 30-39
   - 40-49
   - > 50 anni
2. **In che genere ti identifichi?***
   - M
   - F
   - Altro
3. **Dove sei residente?***
   - Nord
   - Centro
   - Sud Italia o Isole

Sezione 1 - Diagnosi della malattia

4. **A che età hai ricevuto la diagnosi di malattia di Crohn?***
   - 1-9
   - 10-19
   - 20-29
   - 30-39
   - 40-49
   - Sopra i 50 anni

5. **Che tipologia di malattia di Crohn ti è stata indicata al momento della diagnosi?***
Seleziona tutte quelle che si applicano:
   - Ileale
   - Colico
   - Gastro-duodenale
   - Perianale

6. **Quali sintomi riscontri attualmente?***
   Seleziona tutti quelli che si applicano:
   - Dolore addominale
   - Diarrea
   - Fatigue (Senso di stanchezza, eccessiva spossatezza o debolezza, percepita come inusuale o anormale)
   - Nausea
   - Vomito
   - reflusso gastroesofageo
   - Perdita di peso
   - Sanguinamento rettale
   - Dolori articolari

6a. **Se hai selezionato "fatigue", ti senti che è una sensazione persistente?** (domanda facoltativa)

- Sì
- No

6b. **Provi fatigue solo quando la malattia è in fase acuta o anche quando è in remissione?** (domanda facoltativa)

- Sì
- No

7. **Assumi un farmaco per la malattia di Crohn? ***
   - Sì
   - No

7a. **Se sì, di che tipo di farmaco si tratta?***

- Steroideo
- Biologico
- Altro (specificare)

Sezione 2 – Il ruolo della nutrizione

8. **Quanto ritieni importante la nutrizione nella gestione della malattia? Credi che la dieta possa influenzare l'attività della tua malattia?***
   - 1 – molto poco
   - 2 – poco
   - 3 – indifferente
   - 4 – molto
   - 5 – moltissimo

9. **Il tuo gastroenterologo ti ha informato del fatto che la dieta influenza l'attività di malattia?***
   - Sì
   - No

10. **Il tuo gastroenterologo ti ha mai consigliato una visita da un nutrizionista?***
    - Sì
    - No

10.a **Se sì, in ospedale?***

- Sì
- No

10.b **Se no, sei andato da un nutrizionista privatamente?***

- Sì
- No

11. **Nel centro in cui sei in cura è disponibile un dietista/nutrizionista con un accesso agevolato?***
    - Sì
    - No

12. **Ti hanno mai consigliato una dieta per il Crohn?***

- Sì
- No

13. **Ti hanno mai consigliato un supplemento nutrizionale?***
    - Sì
    - No

    13.a **Che tipologia di supplemento ti hanno consigliato?***
    - Supplemento specifico per malattia di Crohn
    - Supplemento nutrizionale ipercalorico e/o iperproteico
    - Integratore vitaminico
    - Integratori di omega 3

    13.b **Se hai risposto "Supplemento specifico per malattia di Crohn", quale?**
    ……
    …….

Sezione 3 – Fase di remissione. Gestione quotidiana e impatto sociale

14. **Durante la tua giornata tipo qual è il pasto più "complicato" da organizzare?* Seleziona una sola opzione:**
    - Colazione
    - Pranzo
    - Merenda
    - Cena
15. **Perché?**
    ……………………………………………………………………

16. **Hai mai dovuto rinunciare ad occasioni sociali (cene, pranzi aziendali, uscite con amici) a causa della malattia? ***
    - Sì
    - No

17. **Segui una dieta particolare per la malattia di Crohn?***
    - Sì
    - No

18. **Conosci la Dieta di Esclusione della Malattia di Crohn (CDED)? ***
    - Sì
    - No

19. **Quale dieta segui? ***
    - Dieta CDED
    - Dieta a basso contenuto di fibra
    - Nessun tipo di dieta
    - Altro (specificare)

Sezione 4 – Fase attiva. Gestione quotidiana e impatto sociale

20. **Durante la tua giornata tipo qual è il pasto più "complicato" da organizzare?* Seleziona una sola opzione:**
    - Colazione
    - Pranzo
    - Merenda
    - Cena

21. **Perché?**
    ……………………………………………………………

22. **Hai mai dovuto rinunciare ad occasioni sociali (cene, pranzi aziendali, uscite con amici) a causa della malattia? ***
    - Sì
    - No

23. **Segui una dieta particolare per la malattia di Crohn?***
    - Sì
    - No

24. **Se si, quale dieta segui? ***
    - Dieta CDED
    - Dieta a basso contenuto di fibra
    - Nessun tipo di dieta
    - Altro (specificare)

Sezione 5 – Sfera psicologica

25. **Ti capita di sentirti in ansia a causa della malattia quando trascorri la giornata fuori casa, sul luogo di lavoro o in università?***
    - Sì
    - No

26. **La malattia ti limita nella scelta delle attività da svolgere nel tempo libero, come ad esempio nello svolgere attività sportiva o altri hobby? ***
    - Sì
    - No

27. **Indica, in ordine di importanza, i sentimenti che provi più spesso in relazione alla malattia. ***
    Seleziona tutti quelli che si applicano:
    - Ansia
    - Stress
    - Imbarazzo
    - Frustrazione
    - Solitudine (senso di esclusione)

28. **Quanto del tuo tempo libero dedichi per seguire il regime alimentare più idoneo per te?* (es. spesa/cucinare/informarti/monitorare i sintomi…):**
    - Meno di 2 ore a settimana
    - Da 2 a 4 ore a settimana
    - Più di 4 ore a settimana

- Più di 6 ore a settimana

29. **Quali sono le tue fonti di informazioni sulla malattia?***
Seleziona tutte quelle che utilizzi:
    - Medico
    - Online
    - Social
    - Associazione pazienti

30. **Quali informazioni ti piacerebbe ricevere? ***

……….

2. Questionnaire (English version)

Respondent Profile

1. **What is your age group?**
    - 1–9
    - 10–19
    - 20–29
    - 30–39
    - 40–49
    - Over 50

2. **What is your gender identity?**
    - Male
    - Female
    - Other

3. **Where do you live?**
    - Northern Italy
    - Central Italy
    - Southern Italy or Islands

Section 1 – Disease Diagnosis

4. **At what age were you diagnosed with Crohn's disease?**
    - 1–9
    - 10–19
    - 20–29
    - 30–39
    - 40–49
    - Over 50

5. **What type of Crohn's disease were you diagnosed with?**
Select all that apply:
    - Ileal
    - Colonic
    - Gastro-duodenal

- Perianal

6. **What symptoms are you currently experiencing?**
   Select all that apply:
   - Abdominal pain
   - Diarrhea
   - Fatigue (feeling of tiredness, excessive exhaustion or weakness perceived as unusual or abnormal)
   - Nausea
   - Vomiting
   - Gastroesophageal reflux
   - Weight loss
   - Rectal bleeding
   - Joint pain

   6a. **If you selected "fatigue", does it feel persistent?** (optional)
   - Yes
   - No

   6b. **Do you experience fatigue only during flares or also in remission?** (optional)
   - Yes
   - No

7. **Are you currently taking medication for Crohn's disease?**
   - Yes
   - No

   7a. **If yes, what type of medication?**
   - Steroids
   - Biologics
   - Other (please specify)

Section 2 – The Role of Nutrition

8. **How important do you consider nutrition in managing your disease? Do you think diet can influence disease activity?**
   - 1 – Not at all
   - 2 – Slightly
   - 3 – Neutral
   - 4 – Very
   - 5 – Extremely

9. **Has your gastroenterologist informed you that diet may influence disease activity?**
   - Yes
   - No

10. **Has your gastroenterologist ever recommended a consultation with a nutritionist?**
   - Yes
   - No

   10a. **If yes, was it in the hospital?**

- Yes
- No

10b. **If not, have you consulted a private nutritionist on your own?**

- Yes
- No

11. **Is there an easily accessible dietitian/nutritionist at your treatment center?**
    - Yes
    - No

12. **Have you ever been advised to follow a specific diet for Crohn's disease?**
    - Yes
    - No

13. **Have you ever been recommended a nutritional supplement?**
    - Yes
    - No

13a. **What type of supplement was recommended?**

- Specific supplement for Crohn's disease
- High-calorie and/or high-protein nutritional supplement
- Vitamin supplement
- Omega-3 supplement

13b. **If you selected "specific supplement for Crohn's disease", which one?**

…………………………………………………………………

Section 3 – Remission Phase: Daily Management and Social Impact

14. **During your typical day, which meal is the most challenging to manage?**
    Select one:
    - Breakfast
    - Lunch
    - Snack
    - Dinner

15. **Why?**
    ……………………………………………………………

16. **Have you ever had to skip social occasions (dinners, work lunches, outings with friends) due to your disease?**
    - Yes
    - No

17. **Are you following a specific diet for Crohn's disease?**
    - Yes
    - No

18. **Are you familiar with the Crohn's Disease Exclusion Diet (CDED)?**
    - o Yes
    - o No

19. **Which diet are you currently following?**
    - o CDED
    - o Low-fiber diet
    - o No specific diet
    - o Other (please specify)

Section 4 – Active Disease Phase: Daily Management and Social Impact

20. **During your typical day, which meal is the most challenging to manage?**
    Select one:
    - o Breakfast
    - o Lunch
    - o Snack
    - o Dinner

21. **Why?**
    …………………………………………………………

22. **Have you ever had to skip social occasions (dinners, work lunches, outings with friends) due to your disease?**
    - o Yes
    - o No

23. **Are you following a specific diet for Crohn's disease?**
    - o Yes
    - o No

24. **If yes, which diet are you currently following?**
    - o CDED
    - o Low-fiber diet
    - o No specific diet
    - o Other (please specify)

Section 5 – Psychological Impact

25. **Do you ever feel anxious due to your disease when spending the day away from home, at work, or at university?**
    - o Yes
    - o No

26. **Does the disease limit your choice of leisure activities, such as sports or other hobbies?**
    - o Yes
    - o No

27. **Which emotions do you most often associate with your disease?**
Select all that apply and rank in order of importance:
  - o Anxiety
  - o Stress
  - o Embarrassment
  - o Frustration
  - o Loneliness (feeling of exclusion)

28. **How much of your free time do you dedicate to maintaining a suitable diet?** (e.g., grocery shopping, cooking, researching, symptom tracking)
  - o Less than 2 hours per week
  - o 2–4 hours per week
  - o More than 4 hours per week
  - o More than 6 hours per week

29. **What are your main sources of information about the disease?**
Select all that apply:
  - o Doctor
  - o Online
  - o Social media
  - o Patient associations

30. **What kind of information would you like to receive?**
…………………………………………………………

3. Supplementary table. CROSS checklist

Section/topic	Item	Item description	Reported on page #
**Title and abstract**			
Title and abstract	1a	State the word "survey" along with a commonly used term in title or abstract to introduce the study's design.	1
	1b	Provide an informative summary in the abstract, covering background, objectives, methods, findings/results, interpretation/discussion, and conclusions.	1
**Introduction**			
Background	2	Provide a background about the rationale of study, what has been previously done, and why this survey is needed.	1,2
Purpose/aim	3	Identify specific purposes, aims, goals, or objectives of the study.	2
**Methods**			
Study design	4	Specify the study design in the methods section with a commonly used term (e.g., crosssectional or longitudinal).	2
Data collection methods	5a	Describe the questionnaire (e.g., number of sections, number of questions, number and names of instruments used).	2
	5b	Describe all questionnaire instruments that were used in the survey to measure particular concepts. Report target population, reported validity and reliability information, scoring/classification procedure, and reference links (if any).	2
	5c	Provide information on pretesting of the questionnaire, if performed (in the article or in an online supplement). Report the method of pretesting, number of times questionnaire was pre-tested, number and demographics of participants	NA

		used for pretesting, and the level of similarity of demographics between pre-testing participants and sample population.	
	5d	Questionnaire if possible, should be fully provided (in the article, or as appendices or as an online supplement).	Yes, in Data Suppl.
Sample characteristics	6a	Describe the study population (i.e., background, locations, eligibility criteria for participant inclusion in survey, exclusion criteria).	2,3,4
	6b	Describe the sampling techniques used (e.g., single stage or multistage sampling, simple random sampling, stratified sampling, cluster sampling, convenience sampling). Specify the locations of sample participants whenever clustered sampling was applied.	NA
	6c	Provide information on sample size, along with details of sample size calculation.	NA
	6d	Describe how representative the sample is of the study population (or target population if possible), particularly for population-based surveys.	NA
Survey administration	7a	Provide information on modes of questionnaire administration, including the type and number of contacts, the location where the survey was conducted (e.g., outpatient room or by use of online tools, such as SurveyMonkey).	2
	7b	Provide information of survey's time frame, such as periods of recruitment, exposure, and follow-up days.	2
	7c	Provide information on the entry process:<br>–>For non-web-based surveys, provide approaches to minimize human error in data entry.<br>–>For web-based surveys, provide approaches to prevent "multiple participation" of participants.	NA
Study preparation	8	Describe any preparation process before conducting the survey (e.g., interviewers' training process, advertising the survey).	NA
Ethical considerations	9a	Provide information on ethical approval for the survey if obtained, including informed consent, institutional review board [IRB] approval, Helsinki declaration, and good clinical practice [GCP] declaration (as appropriate).	NA
	9b	Provide information about survey anonymity and confidentiality and describe what mechanisms were used to protect unauthorized access.	2
Statistical analysis	10a	Describe statistical methods and analytical approach. Report the statistical software that was used for data analysis.	NA
	10b	Report any modification of variables used in the analysis, along with reference (if available).	NA
	10c	Report details about how missing data was handled. Include rate of missing items, missing data mechanism (i.e., missing completely at random [MCAR], missing at Random [MAR] or missing not at random [MNAR]) and methods used to deal with missing data (e.g., multiple imputation).	NA
	10d	State how non-response error was addressed.	NA
	10e	For longitudinal surveys, state how loss to follow-up was addressed.	NA
	10f	Indicate whether any methods such as weighting of items or propensity scores have been used to adjust for non-representativeness of the sample.	NA
	10g	Describe any sensitivity analysis conducted.	NA

Results

Respondent characteristics	11a	Report numbers of individuals at each stage of the study. Consider using a flow diagram, if possible.	3,4,5,6,7
	11b	Provide reasons for non-participation at each stage, if possible.	NA
	11c	Report response rate, present the definition of response rate or the formula used to calculate response rate.	NA
	11d	Provide information to define how unique visitors are determined. Report number of unique visitors along with relevant proportions (e.g., view proportion, participation proportion, completion proportion).	NA
Descriptive results	12	Provide characteristics of study participants, as well as information on potential confounders and assessed outcomes.	2,3
Main findings	13a	Give unadjusted estimates and, if applicable, confounder-adjusted estimates along with 95% confidence intervals and p-values.	NA
	13b	For multivariable analysis, provide information on the model building process, model fit statistics, and model assumptions (as appropriate).	NA
	13c	Provide details about any sensitivity analysis performed. If there are considerable amount of missing data, report sensitivity analyses comparing the results of complete cases with that of the imputed dataset (if possible).	NA

Discussion

Limitations	14	Discuss the limitations of the study, considering sources of potential biases and imprecisions, such as non-representativeness of sample, study design, important uncontrolled confounders.	10
Interpretations	15	Give a cautious overall interpretation of results, based on potential biases and imprecisions and suggest areas for future research.	8,9,10,11
Generalizability	16	Discuss the external validity of the results.	8,9,10,11

Other sections

Role of funding source	17	State whether any funding organization has had any roles in the survey's design, implementation, and analysis.	11
Conflict of interest	18	Declare any potential conflict of interest.	11
Acknowledgements	19	Provide names of organizations/persons that are acknowledged along with their contribution to the research.	11
